# TRPA1 as a Key Regulator of Keratinocyte Homeostasis and Inflammation in Human Skin

**DOI:** 10.3390/cells15020192

**Published:** 2026-01-20

**Authors:** Caterina Cattani, Claudia Scarponi, Martina Morelli, Kilian Eyerich, Stefanie Eyerich, Christian Napoli, Stefania Madonna, Cristina Albanesi, Andrea Cavani, Fernanda Scopelliti

**Affiliations:** 1 National Institute for Health, Migration and Poverty INMP/NIHMP, via di S. Gallicano, 25, 00153 Rome, Italy; caterina.cattani@inmp.it (C.C.); f.scopelliti@inmp.it (F.S.); 2 Laboratory of Experimental Immunology, Isituto Dermatopatico Italiano IDI-IRCCS, via Monti di Creta, 04, 00167 Rome, Italy; c.scarponi@idi.it (C.S.); s.madonna@idi.it (S.M.);; 3 Clinic of Dermatology and Venereology, Medical Center-University of Freiburg, Faculty of Medicine, University of Freiburg, 79104 Freiburg, Germany

**Keywords:** TRP, transient receptors, keratinocytes, skin

## Abstract

The Transient Receptor Potential Ankyrin 1 (TRPA1) channel is a non-selective cation channel activated by a range of physical and chemical stimuli. While primarily studied in neuronal tissues, TRPA1 is also expressed in human keratinocytes, where its role remains poorly understood. Here, we investigated TRPA1 expression and function in keratinocytes and examined the effects of its activation on cellular proliferation, immune activation, and neuropeptide release under both basal and inflammatory stimuli. TRPA1 expression was detected in basal keratinocytes and was upregulated by pro-inflammatory cytokines. Stimulation with the TRPA1 agonist allyl isothiocyanate (AITC) induced a rapid calcium influx, confirming functional channel activity. AITC at 5 µM did not induce cytotoxicity but significantly reduced keratinocyte proliferation and caused cell cycle arrest. Under stimulation with TNF-α and IFN-γ, TRPA1 activation decreased the surface expression of HLA-DR and ICAM-1, and downregulated mRNA levels of CXCL10, CXCL8, CCL5, and CCL20, while IL-6 expression remained unchanged. Furthermore, AITC treatment reduced the secretion of Substance P, but not CGRP. These findings indicate that TRPA1 functions as a cytokine-inducible, immunomodulatory receptor in human keratinocytes, capable of attenuating proliferation and inflammatory activation without compromising cell viability, thereby suggesting a potential role in maintaining skin homeostasis and modulating cutaneous inflammation.

## 1. Introduction

The Transient Receptor Potential Ankyrin 1 (TRPA1) channel is a calcium-permeable member of the TRP ion channel superfamily. Classification of TRP channels is based on amino acid sequence similarities and includes multiple subfamilies, such as TRPC, TRPV, TRPM, TRPML, TRPP, and TRPA [1,2].

TRPA1 responds to a diverse array of endogenous and exogenous stimuli, including noxious temperatures (e.g., temperatures below 17 °C) and cellular stress and damage, as well as various pungent and chemical compounds such as isothiocyanates, formaldehyde, mustard oil, cinnamaldehyde, gingerol, carvacrol (from oregano), allicin (from garlic), eugenol (from clove oil), and methyl salicylate (from wintergreen oil) [3,4,5,6].

TRP members were initially discovered in neuronal cells, where their activation triggers the release of neurotransmitters, including substance P (SP) and calcitonin gene-related peptide (CGRP) [7]; more recently, the channel has been identified in other cell types, such as keratinocytes, fibroblasts, melanocytes, endothelial cells, and immune cells [8,9,10].

In the skin, TRPA1 functions as a mediator of nociception, responding to noxious stimuli such as irritants, mechanical stress, and temperature changes. The epidermis exhibits a gradient of calcium concentration, which plays a central role in the regulation of keratinocyte differentiation. As keratinocytes migrate towards the surface of the epidermis, the calcium concentration increases, triggering differentiation processes that culminate in the formation of the stratum corneum, the outermost protective layer of the skin. TRPA1-mediated calcium influx is thought to be involved in this process, facilitating the transition from proliferation to differentiation of keratinocytes [11]. Furthermore, melanocyte exposure to UVA light activates TRPA1 through reactive oxygen species, triggering the initial phase of the biphasic hyperpigmentation response. This implies that TRPA1 is involved in the skin’s reaction to UV light and potentially in pigmentation processes [12].

TRPA1, leading to the release of pro-inflammatory mediators and neuropeptides, has been proposed to contribute to pain, inflammation, and itching [13,14,15,16]. Recent studies also suggest that TRPA1 is involved in the regulation of skin barrier function and wound healing because enhances the release of growth factors and cytokines that promote cell proliferation, migration, and tissue remodeling [17,18,19]. This contributes to the regeneration of the epidermis and restoration of skin integrity, protecting the body from environmental insults and preventing dehydration.

TRPA1’s dual role in both promoting wound healing and exacerbating inflammation may be context-dependent, and it remains a subject of ongoing investigation to determine its precise contribution to skin disease pathophysiology. In inflammatory skin disorders, such as atopic dermatitis and psoriasis, TRPA1 is thought to contribute to pathological processes. However, it also appears to play a protective role by alleviating symptoms in mouse models [20,21]. Given these complexities, our study aims to fully elucidate the role of TRPA1 in healthy skin, focusing on its dual function in promoting keratinocyte differentiation, regulating skin barrier function, and mediating inflammation. By addressing this knowledge gap, we hope to deepen our understanding of TRPA1’s intricate and controversial role in skin biology, offering insights into its potential as a therapeutic target in both chronic skin diseases and regenerative therapies.

## 2. Material and Methods

### 2.1. Ethics Statement

The study conformed to the ethical principles for medical research involving human participants outlined in the Declaration of Helsinki and was approved by the Ethics Committee of IDI-IRCCS, Rome, Italy (Prot. 581-2/2019, approved on 15 July 2019). Participation was voluntary. Informed consent was obtained from all subjects involved in the study.

### 2.2. Human Materials

Healthy skin samples were obtained from donors undergoing abdominoplasty surgery (*n* = 3; two females and one male, aged 40–60 years). Skin biopsies were used for immunohistochemistry (IHC) and for the isolation of primary human keratinocyte cultures [22].

### 2.3. Immunohistochemistry (IHC)

Paraffin-embedded sections were obtained from biopsies of healthy skin. Five-μm sections were dewaxed and rehydrated. After quenching endogenous peroxidase, achieving antigen retrieval and blocking non-specific binding sites was performed as indicated by the manufacturer (Vector Laboratories, Newark, CA, USA). Sections were incubated with anti-human TRPA1 (extracellular) rabbit polyclonal antibody (ACC-037, Alomone Lab, Jerusalem, Israel), at a concentration of 4 μg/mL. Secondary staining kits were obtained from Vector Laboratories. Immunoreactivity was visualized using DAB and specimen counterstained with hematoxylin. As a negative control, blocking peptide at a concentration of 20 μg/mL was used. Stained sections were analyzed with axioplan 2 (Zeiss, Oberkochen, Germany).

### 2.4. Keratinocyte Cultures and Treatments

Second- or third-passage keratinocytes were used in all experiments, with cells cultured in serum-free keratinocyte growth medium (KGM, Clonetics, San Diego, CA, USA) for at least 3–5 days (at 60–80% confluence), before performing cytokine treatment. For the treatments with TRPA1 agonists (AITC, JT010, ASP7663), preliminary experiments aimed at identifying the optimal dose (in terms of efficacy and cytotoxicity) for each of them were performed. Specifically, 1, 5, and 10 μM concentrations were tested for AITC, 1 and 5 μM for JT, and 2 and 5 μM for ASP7663. Cytotoxicity of AITC, JT, and ASP7663 was tested by measuring the lactate dehydrogenase (LDH) released from keratinocyte cultures, using cytotoxicity Detection Kit Plus-LDH (Roche Diagnostics, Milan, Italy), following the manufacturer’ instructions. Thus, keratinocyte cultures were treated with the optimal dose of AITC (5 μM), JT010 (5 μM), and ASP7663 (2 μM). Cytokine stimulations with recombinant human (rh) IFN-γ (200 U/mL), TNF-α (50 ng/mL), and IL-17A (50 ng/mL) (R&D Systems, Minneapolis, MN, USA), as well as treatments with TRPA1 agonists, were conducted on keratinocyte cultures kept in keratinocyte basal medium (KBM-GOLD, Clonetics, Lonza, Basel, Switzerland).

### 2.5. Fura Red Analysis

Keratinocytes were resuspended at 1 × 10^7^ cells/mL in 37 °C PBS with 1 μM of Fura Red AM (Invitrogen, Paisley, UK) and incubated at 37 °C for 30 min. Cells were washed and resuspended to 1 × 10^7^ cells/mL in RPMI with 1% FBS; cells were allowed to equilibrate for 10 min at 37 °C and subsequently analyzed by flow cytometry. Background, non-specific calcium flux was recorded for 240 s; then, the cells were treated with Allyl isothiocyanate (AITC, Sigma Aldrich, St. Louis, MO, USA), 100 μM. Continuous recording rate was 8000–10,000 events/second for 240 s in total. The ratiometric ‘Fura Red Ratio’ was calculated as the increasing signal stimulated by the Violet laser over the decreasing signal stimulated by the Green laser (406 nm/532 nm), using the Kinetics tool in Flow Jo software version 10.8.1 (Tree Star Inc., Ashland, OR, USA).

### 2.6. Flow Cytometry

Keratinocytes treated with different stimuli were washed with PBS and stained with TRPA1 antibody (Alomone) for 20 min followed by anti-rabbit PE (R&D, USA) as a secondary antibody. Mouse IgG isotypes and blocking peptides were used as controls (BD Biosciences, Franklin Lakes, NJ, USA). Keratinocyte expression of membrane ICAM-1 and HLA-DR was evaluated using fluorescein isothiocyanate (FITC)-conjugated anti-CD54 (clone 84H10; Immunotech, Marseille, France) and anti-HLA-DR (clone L243, BD Pharmingen, Franklin Lakes, NJ, USA), Abs. For acquisition and analysis, the first was performed using an Attune Nxt (Life Technologies, Carlsbad, CA, USA) cytofluorimeter or the Accuri C6 Flow cytometer (BD), whereas the second was performed using Flow logic software v10 (Miltenyi, Bergisch Gladbach, Germany) or Cell Quest software v5.1 v10(BD), according to the Guidelines for the use of flow cytometry and cell sorting in immunological studies [23].

### 2.7. Cell Cycle Analysis

To determine cell-cycle distribution analysis, cells were cultured in six-well plates, and, at 60–80% confluence, treated with 5 μM AITC or vehicle alone (0.1% DMSO) in KGM for 24 h. DNA content was detected using a BD Cycletest™ Plus DNA kit (BD). Briefly, after treatment, cells were collected by trypsinization and washed three times with kit buffer solution. Following sequential incubations with Solution A (trypsin buffer), Solution B (trypsin inhibitor and RNase buffer), and Solution C (PI stain solution), keratinocytes DNA content was analyzed by using an Accuri C6 Flow cytometer (BD) equipped with Cell Quest software (BD).

### 2.8. Proliferation Assays

Proliferation of keratinocyte cultures was evaluated by using a CyQuant Cell proliferation Kit (ThermoFisher Scientific, Waltham, MA, USA), which measures total DNA content. Briefly, 0.5–1 × 10^4^ cells were grown for 24, 48, and 72 h in 96-well plates in KBM or KGM, in the presence or absence of 5 μM AITC. Then, cells were stained with CyQUANT dye, whose emission fluorescence was measured at 530 nm using the EnSight multimode plate reader (Perkin Elmer, Waltham, MA, USA).

### 2.9. siRNA (Small Interfering RNA) Transfection

A pool of four siRNA duplexes designed to specifically knockdown TRPA1 (L-006109-02-0005, Dharmacon, ThermoFisher) was used to transfect primary cultures of human keratinocytes (40 nM siRNA/3 cm plate) using INTERFERin transfectant (PolyPlus, Sartorius, Gottingen, Germany), following the manufacturer’s protocol. Non-targeting negative control siRNA (D-001810-10, Dharmacon, ThermoFisher) was used to control non-specific effects of the transfection. After a 48 h transfection, cells were left untreated or treated with IFN-γ and TNF-α for 8 h for the further real-time PCR and Western blotting analyses.

### 2.10. RNA Isolation and Real-Time Polymerase Chain Reaction (PCR)

Total RNA from treated keratinocytes was extracted using the TRIzol reagent (Invitrogen), and mRNA was reverse-transcribed into cDNA and analyzed by real-time PCR. The expression of suppressor of CXCL10, CCL5, CCL20, IL-6, CXCL8 and HPRT1 mRNA was evaluated by Quant Studio 5 (ThermoFisher), using SYBR Green PCR Master Mix. The forward and reverse primers employed for real-time PCR were as follows: for CXCL10, Fw 5′-TCT GAG TGG GAC TCA AGG GAT-3′ and Rev 5′-ATT CTC ACT GGC CCG TCA TC-3′, for CCL5, Fw 5′-CCC ACA AGA GGA CTC ATT CCA A-3′ and Rev 5′-TTG ATC TGA GCT GGG CAT TG-3′, for CCL20, Fw 5′GTG CTG CTA CTC CAC CTC TG-3′ and Rev 5′ TGT ATC CAA GAC AGC AGT CAA A-3′, for IL-6, Fw 5′-GGC ACT GGC AGA AAA CAA CC-3′ and Rev 5′-CAC CAG GCA AGT CTC CTC AT-3′, for CXCL8, Fw 5′-CCC CTA AGA GCA GTA ACA GTT CCT-3′ and Rev 5′-GGT GAA GAT AAG CCA GCC AAT C-3′,CCL2 Fw 5′- CAC CAG CAG CAA GTG TCC C CCL2 Rev 5′CCA TGG AAT CCT GAA CCC ACTRPA1 Fw 5′-CAC CTG GCT GTG CAA AAT GG TRPA1 Rev 5′ CTG TGC ACC TTC CCT TCT CC. The levels of gene expression were determined by normalizing to HPRT-1 mRNA expression. The values obtained from triplicate experiments were averaged, and data are presented as means 6 SD.

### 2.11. ELISA

Supernatants from Keratinocytes treated with different stimulus in the presence or absence of AITC were collected at 24 h. Supernatant contents of CXCL10, CCL5, CCL20, CCL2, CXCL8, Substance P, and CGPR were measured using commercially available sandwich ELISA kits (all from R&D systems, Minneapolis, MN, USA).

### 2.12. Statistical Analysis

For in vitro studies, statistical significance was evaluated using unpaired t tests performed by Prism v.5.0 (GraphPad Software, La Jolla, CA, USA), and values were expressed as the mean + SD. Values of *p* < 0.05 were considered significant.

## 3. Results

### 3.1. TRPA1 Expression in the Skin

**Figure 1:**
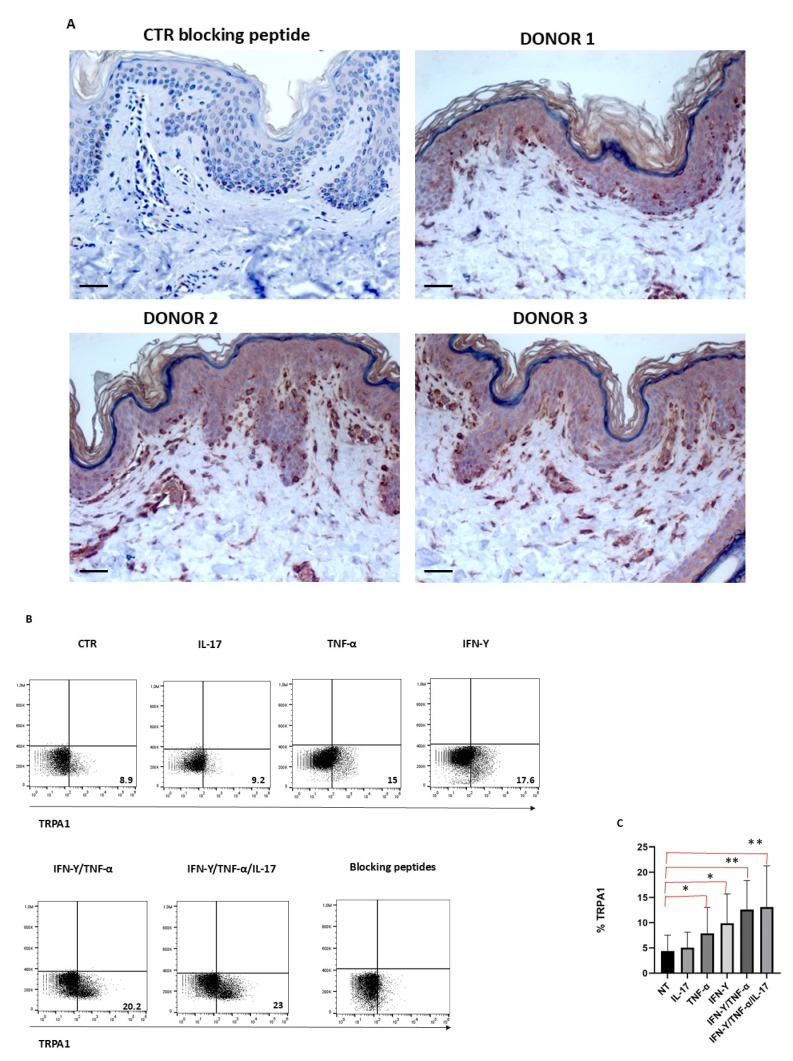
TRPA1 expression in human skin tissues, and in keratinocytes. (**A**) Representative IHC staining of TRPA1 expression (red-brown stained), performed on paraffin-embedded sections of biopsies obtained from healthy donors (n = 3). A magnification of 200× is shown. Negative control (CTR) of TRPA1 staining was obtained by pre-incubating the antibody with the blocking peptide, which completely abolished the signal, confirming the specificity of the staining. One representative TRPA1 staining for the three donors is shown. Scale bars, 50 mm. (**B**) Keratinocytes obtained from three different donors were cultured for 24 h in the presence of different cytokines and analyzed with TRPA1 antibody and blocking peptide by FACS, as described in methods. (**C**) Graphs show the percentage of TRPA1 + cells treated with different inflammatory stimuli from each donor (* *p* < 0.05; ** *p* < 0.005).

To assess the expression of TRPA1 in the skin, immunohistochemistry on skin specimens obtained from healthy donors was performed. As shown in Figure 1A, TRPA1 staining was observed in basal keratinocytes, fibroblasts, nerve endings, and sebaceous glands and cells with a dendritic morphology, presumably Langerhans cells. Cell membrane expression of TRPA1 expression was detected in the skin of all three donors, with varying staining intensities. Notably, strong positivity was observed in basal keratinocytes, suggesting their involvement in skin homeostasis. Importantly, the use of a blocking peptide completely abolished the signal, confirming the specificity of the staining.

### 3.2. Keratinocyte TRPA1 Expression Modulation by Pro-Inflammatory Cytokines

To understand whether TRPA1 expression in keratinocytes is modulated by inflammatory stimuli, cells were cultured in the presence or absence of different inflammatory stimuli and then analyzed by cytofluorimetry. TRPA1 was expressed only by a small percentage of keratinocytes; this percentage increased when cells were treated with IFN-γ and TNF-α, while IL-17 appeared to have no effect on TRPA1 expression. When administered together, TNF-α and IFN-γ had a synergistic effect, significantly increasing TRPA1 expression (Figure 1B,C). Antibody specificity in TRPA1-positive samples was validated through the use of a blocking peptide, demonstrating that the observed immunohistochemical signal was specific.

### 3.3. Functionally Active Membrane-Associated TRPA1 on Keratinocytes

**Figure 2:**
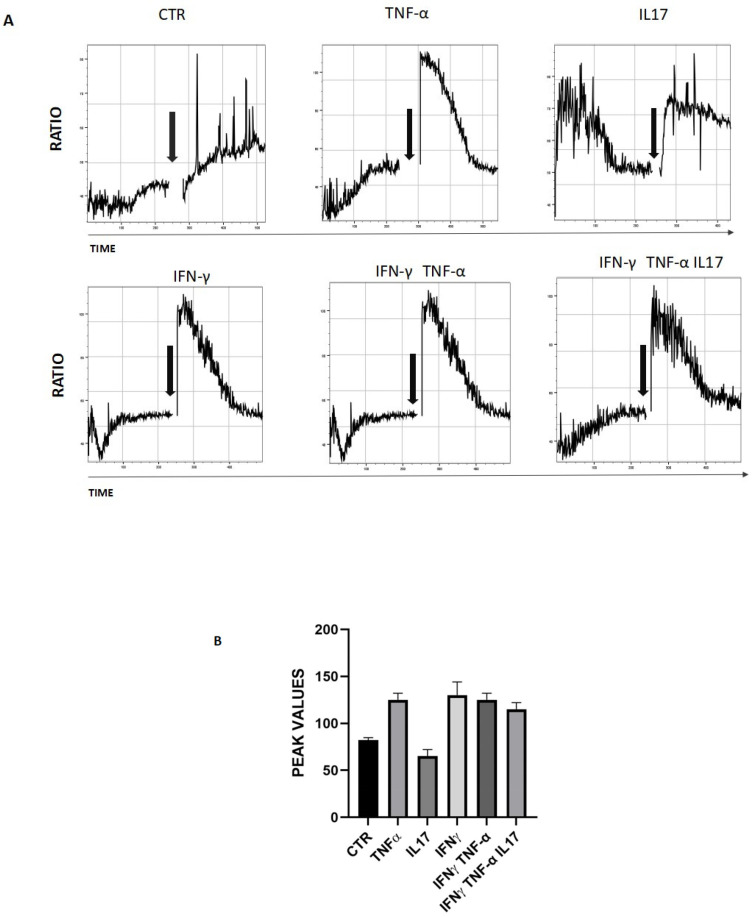
Functional activation of TRPA1 induces intracellular calcium flux in keratinocytes. (**A**) Keratinocytes cells were loaded with Fura Red, AM for 30 min. Intracellular calcium mobilization in response to AITC (100 nM) was measured by flow cytometry. Arrows indicate the addition of substances. Graphs shown the mean value of the Fura Red Ratio over time, in response to AITC. (**B**) Graph of maximum values of the Fura-2 ratio measured at the time of stimulus-dependent activation in three different experiments.

To examine whether TRPA1 channels were functional, calcium flux was measured by Fura Red in freshly isolated keratinocytes pre-treated for 18 h with different cytokines and upon stimulation with the TRPA1 ligand allyl isothiocyanate (AITC). In the presence of extracellular Ca^++^, AITC 100 μM induced a rapid increase in intracellular Ca^++^ in all conditions (Figure 2). The Violet laser detects an increasing fluorescence signal when calcium is released into the cell cytoplasm, whereas the Green laser detects a decreasing signal when fluorescence is quenched by cytoplasmic calcium. Results are calculated as emission from the Violet laser over emission from the Green laser. Our data show that AITC induces a strong increase in calcium released into the cell cytoplasm compared to the control (Figure 2).

### 3.4. TRPA1 Activation Inhibits Keratinocyte Proliferation and Activation

**Figure 3:**
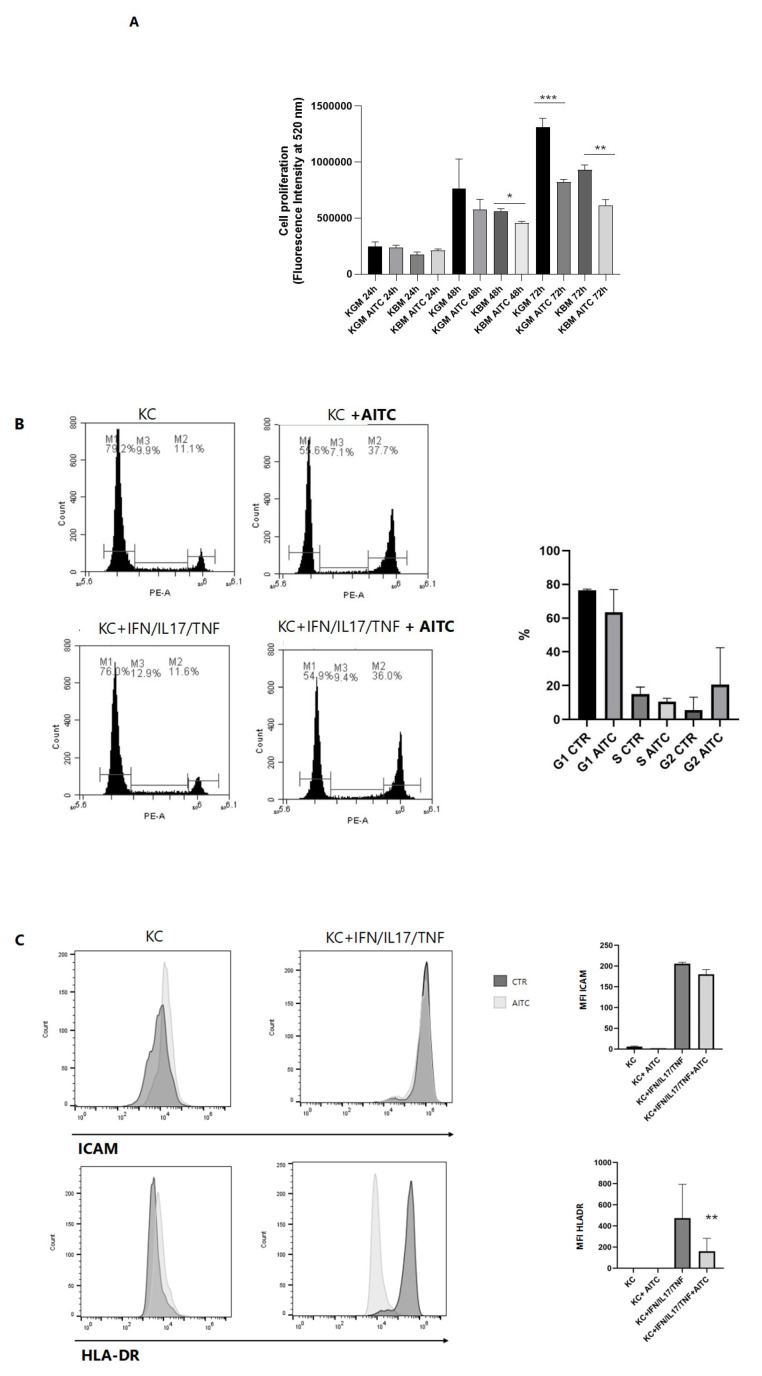
TRPA1 activation suppresses keratinocyte proliferation and activation markers. (**A**) Keratinocytes were cultured in complete Keratinocyte Growth Medium (KGM) or Keratinocyte Basal Medium (KBM), in the presence or absence of AITC (5 mM) for 24, 48, or 72 h. Cells were stained with CyQUANT dye as described in the Methods section. Proliferation is expressed as the mean ± SD of CyQUANT fluorescence intensities, measured at 520 nm in three experiments. * *p* < 0.05, ** *p* < 0.005, *** *p* < 0.0005. (**B**) Keratinocyte cells were treated with different cytokines and AITC, loaded with BD Cycletest™ Plus DNA kit, and analyzed by flow cytometry. Graphs show a representative DNA content histogram. The bars M1, M2, and M3 correspond to different phases of the cell cycle: M1 indicates cells in the G0/G1 phase, M2 represents cells in the S phase, and M3 corresponds to cells in the G2/M phase. Bar graphs show mean percentage values from three experiments (mean ± SD). (**C**) Keratinocytes cells were treated with different cytokines and AITC, and ICAM and HLA-DR receptors were measured by flow cytometry. Bar graphs show mean percentage values from three experiments (mean ± SD).

Keratinocyte cultures were treated with AITC in the presence or absence of inflammatory stimuli. Proliferation assays demonstrated that AITC substantially reduced keratinocyte proliferation both in KBM and KGM complete medium (Figure 3A). Importantly, AITC treatments also induced a cell cycle arrest of keratinocytes in G phases and, in parallel, significantly decreased the percentage of cells in S phases (Figure 3B). In addition to its anti-proliferative effects, AITC treatment led to a marked reduction in HLA-DR expression and a non-significant downregulation of ICAM1 on keratinocytes in the presence of inflammatory stimuli that upregulate the expression of these activation markers (Figure 3C).

### 3.5. TRPA1 Activation Downregulates Keratinocyte Expression of Different Chemokines

**Figure 4:**
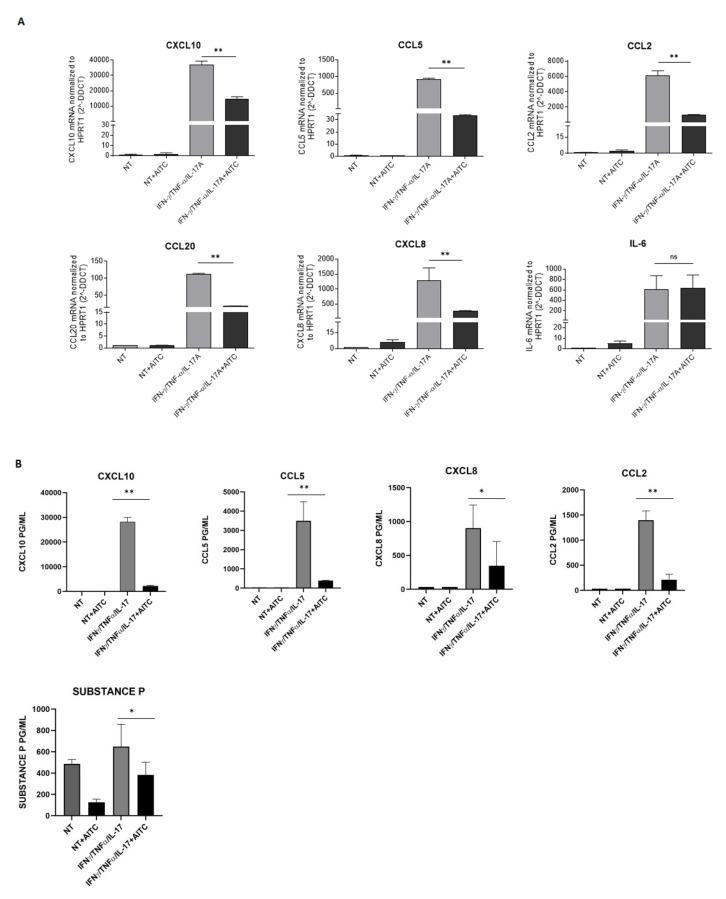
TRPA1 activation downregulates inflammatory chemokines and neuropeptide expression. (**A**) Keratinocytes obtained from three different donors were cultured 18 h in the presence or absence of a mixture of IFN-y and TNF-α alone or in combination with AITC, and analyzed by real-time PCR, as described in the Methods section. (**B**) Cytokine content was measured by ELISA in supernatants of Keratinocytes cultures with different cytokines and treated or untreated with AITC. Data are expressed as mean picograms ± SD of triplicates (* *p* ≤ 0.05 ** *p* ≤ 0.005 as calculated by unpaired Student’s *t* test).

To disclose the role of TRPA1, keratinocytes were treated with AITC, in the presence or absence of a mixture composed of IFN/IL17/TNF, for 18 h, and the expression of different chemokines was analyzed by real-time PCR. As shown in Figure 4A, treatment with 5μM AITC decreased the expression of CXCL10, CCL5, CCL20, and CXCL8, whereas IL6 was substantially unaffected. Notably, the downregulation of these chemokines was further confirmed by ELISA (Figure 4B). To further validate the specificity of AITC-mediated TRPA1 activation, we first evaluated the ability of the other tested TRPA1 agonists—JT010 and ASP7663—to induce intracellular calcium influx in cytokine-treated keratinocytes. Having confirmed that all these compounds effectively increased calcium signaling under inflammatory conditions (Supplementary Figure S1A), additional selective TRPA1 agonists were included in the experimental design to assess their impact on the inflammatory response. In particular, JT010 and ASP7663 were used to evaluate their effects on chemokine expression. These compounds were tested on the three chemokines that showed the strongest downregulation following AITC treatment. Consistently with the effects observed for AITC, both JT010 and ASP7663 reproduced a comparable inhibitory trend, thereby reinforcing the conclusion that TRPA1 activation contributes to the modulation of keratinocyte inflammatory responses. The complete dataset for these experiments is reported in Supplementary Figure S1.

### 3.6. TRPA1 Activation Downregulates Substance P Production

TRPA1 is closely linked to pain because it is a receptor that responds to various noxious stimuli, including irritants, toxins, and environmental factors, which can influence inflammatory responses. For this reason, we aimed to evaluate the expression of substance P in keratinocytes. Keratinocytes were treated with AITC, in the presence or absence of a mixture composed of IFN/IL17/TNF, for 18 h. The results show differences between controls and treated samples: in particular, in the presence of AITC, cells can secrete a reduced amount of Substance P (Figure 4B). Moreover, keratinocytes do not produce CGRP under any of the experimental conditions.

### 3.7. TRPA1 Knockout Modestly Enhances Keratinocyte Expression of Multiple Chemokines

**Figure 5:**
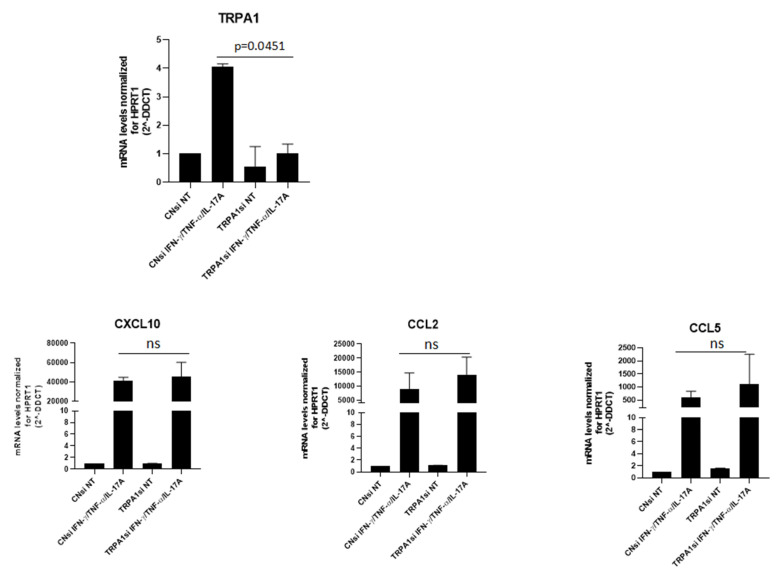
TRPA1 silencing modestly enhances keratinocyte expression of multiple chemokines. Keratinocytes obtained from three different donors were cultured for 18 h in the presence or absence of IFN-γ and TNF-α, in both control cells and silencing-treated cells, and then analyzed by real-time PCR, as described in the Methods section.

To assess whether the modulation of chemokines is selectively linked to TRPA1, gene-silencing experiments were performed. As shown in Figure 5, transfection led to a reduction in TRPA1 expression, which was particularly evident in the presence of cytokines. Silenced cells displayed a slight increase in CXCL10, CCL2, and CCL5 expression, although this change did not reach statistical significance.

## 4. Discussion

In this study, we investigated the expression and functional relevance of the TRPA1 channel in human keratinocytes, providing new insights into its role in skin physiology and immune modulation. Our findings confirm that TRPA1 is functionally expressed in keratinocytes, with predominant localization in the basal layer of healthy epidermis. Notably, its expression is upregulated by pro-inflammatory cytokines such as TNF-α and IFN-γ, suggesting a dynamic regulation of TRPA1 under inflammatory conditions.

While TRPA1 has been extensively characterized in nociceptive neurons for its role in sensing pain, itching, and temperature, growing evidence indicates its involvement in non-neuronal tissues, including skin. Consistent with this broader functional landscape, our data highlight TRPA1’s participation in keratinocyte-mediated responses that go beyond nociception, encompassing cellular proliferation, differentiation, and immune signaling. TRPA1 is also expressed in various immune cells, including macrophages, dendritic cells, and NK and T lymphocytes, further supporting its role in immune modulation and skin homeostasis [24].

Under physiological conditions, TRPA1 expression in keratinocytes is limited and largely confined to the basal layer. In line with previous studies [25], we observed that inflammatory stimuli such as TNF-α and IFN-γ upregulate TRPA1 expression, indicating a possible involvement in the modulation of the inflammatory response. This suggests that TRPA1 may act as a critical sensor for inflammatory states, modulating the skin’s reaction to immune mediators.

Basal keratinocytes proliferate to sustain the continuous renewal of the epidermis, enabling the upward migration of differentiating cells—a process critically dependent on intracellular calcium signaling. In our study, activation of TRPA1 with a specific agonist (AITC) facilitated calcium influx in keratinocytes, as also observed in other cellular models [8,26,27]. This calcium entry plays a central role in regulating multiple calcium-dependent processes, including keratinocyte proliferation, differentiation, and the release of cytokines and chemokines.

The elevated intracellular Ca^2+^ levels triggered by TRPA1 activation appear to promote keratinocyte differentiation and their transition from the basal to the suprabasal layers, a key event for proper epidermal stratification. This differentiation cascade is essential for skin barrier renewal and for shielding underlying tissues from environmental insults [28]. Our results further support the role of TRPA1 in maintaining epidermal homeostasis, aligning with previous evidence that implicates this channel in the regulation of skin barrier integrity [29].

Importantly, in addition to its effects on calcium signaling, AITC exerted a marked antiproliferative effect on keratinocytes. Cell proliferation assays revealed a significant decrease in keratinocyte growth following AITC treatment, supporting the hypothesis that TRPA1 activation limits excessive cellular proliferation. Flow cytometric analysis further showed that AITC induces cell cycle arrest at the G2/M phase, suggesting that calcium influx via TRPA1 interferes with cell cycle progression. The absence of increased LDH release at the tested concentrations excludes non-specific cytotoxicity, indicating a specific regulatory mechanism.

Moreover, AITC treatment resulted in a significant downregulation of HLA-DR expression and a slight decreasing trend of ICAM-1, although the latter did not reach statistical significance. Since HLA-DR and ICAM-1 are involved in antigen presentation and immune cell trafficking, respectively, their reduction suggests that TRPA1 activation may impair keratinocyte-mediated immune activation, thus promoting a more tolerogenic or anti-inflammatory environment.

Further supporting this, TRPA1 activation led to decreased expression of key chemokines such as CXCL10, CCL5, CCL20, and CXCL8, while IL-6 levels remained largely unaffected. These chemokines are known to recruit T cells, neutrophils, and other immune cells to inflamed skin; their downregulation may contribute to the attenuation of local inflammation.

TRPA1 knockdown resulted in a modest upward trend in the expression of all three chemokines; however, these changes did not reach statistical significance. The limited magnitude of the effect is likely related to the complexity of the silencing approach in this experimental system. TRPA1 is poorly expressed in keratinocytes under basal conditions and is strongly induced only upon stimulation with pro-inflammatory cytokines. Under these conditions, achieving complete TRPA1 silencing is technically challenging, and residual receptor expression is expected to persist, potentially dampening the functional impact of knockdown. The observed trend toward a slight increase in chemokine expression may reflect compensatory or redundant regulatory mechanisms within the inflammatory signaling network of keratinocytes. Importantly, our findings are consistent with recent published data [30], which also report no robust effects of TRPA1 modulation on chemokine expression.

Additionally, it is important to note that AITC has been reported to reduce the production of substance P, a neuropeptide involved in pain and inflammation. Previous work has shown that reduced substance P, via activation of other TRP channels, is associated with improved wound healing [31]. The suppression of substance P following TRPA1 activation may thus represent another mechanism through which this channel modulates inflammatory responses, with potential implications for the treatment of chronic inflammatory conditions such as psoriasis.

Beyond inflammatory signaling, other physiological and environmental factors may also contribute to TRPA1 upregulation and activation in basal keratinocytes in vivo. Mechanical stress and tissue stretching can modulate ion channel expression in the epidermis and may similarly influence TRPA1. Epidermal barrier disruption is another relevant stimulus: changes in calcium gradients, release of alarmins, and altered lipid composition could indirectly enhance TRPA1 expression or sensitization. Neurogenic factors—such as neuropeptides released by cutaneous sensory fibers—may further modulate TRPA1 activity. Finally, exposure to environmental irritants or reactive chemicals can activate TRPA1 directly, potentially reinforcing its expression through feed-forward mechanisms. These non-inflammatory pathways may act in parallel with immune signals and together shape TRPA1 regulation in basal keratinocytes.

In conclusion, our findings identify TRPA1 expression in a specific subset of keratinocytes and demonstrate its functional involvement in the regulation of keratinocyte biology and inflammatory signaling. We show that TRPA1 is upregulated by pro-inflammatory cytokines and directly influences keratinocyte proliferation, differentiation, and chemokine production, supporting its role in maintaining epidermal homeostasis. These results suggest that TRPA1 may represent a potential therapeutic target in inflammatory skin conditions. Further investigation is nonetheless required to define the molecular pathways controlling TRPA1 activity in psoriasis and to clarify its contribution to epidermal immune regulation and barrier integrity.

## Data Availability

The original contributions presented in this study are included in the article/Supplementary Material. Further inquiries can be directed to the corresponding author.

## Supplementary Materials

The following supporting information can be downloaded at https://www.mdpi.com/article/10.3390/cells15020192/s1.
