# Supplementary figures and images for: TRPA1 as a Key Regulator of Keratinocyte Homeostasis and Inflammation in Human Skin
Source: Cells. 2026 Jan 20;15(2):192. doi: 10.3390/cells15020192 (PMC12840384; doi:10.3390/cells15020192)

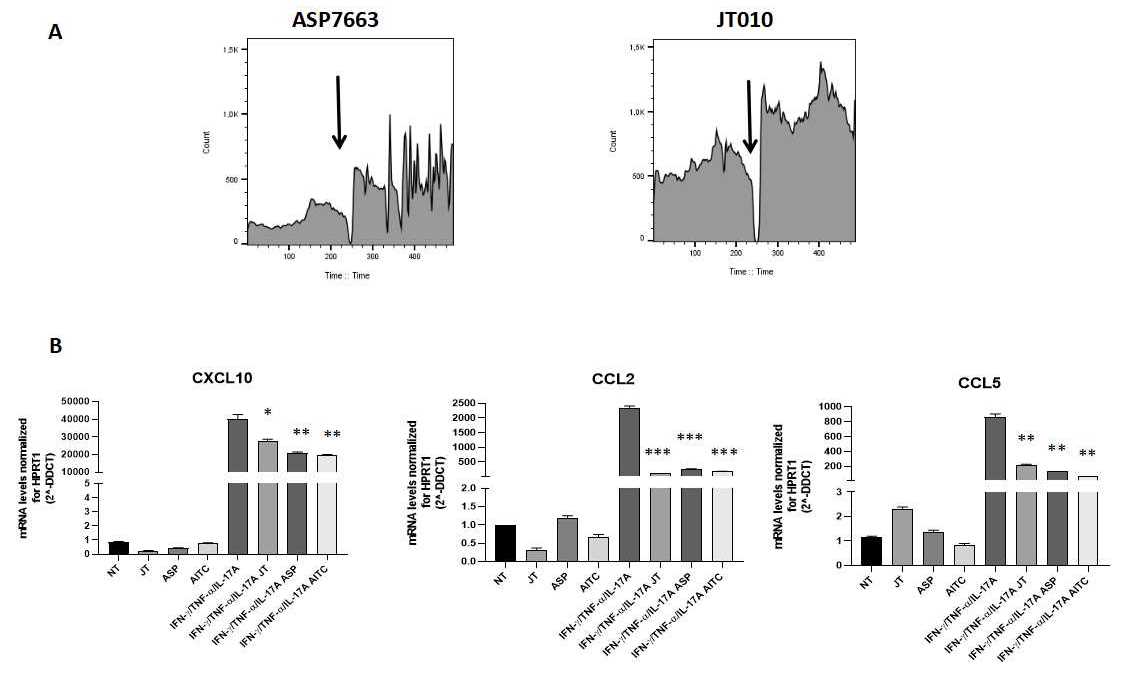

Supplement: Supplementary file 1 [file cells-15-00192-s001.zip › Supplementary figure 1.jpg]
